# Supplementary material for: The Relationships between Caregiver Burden, Physical Frailty, Race, Behavioral and Psychological Symptoms (BPSD), and Other Associated Variables: An Exploratory Study
Source: Medicina (Kaunas). 2024 Mar 1;60(3):426. doi: 10.3390/medicina60030426 (PMC10972283; doi:10.3390/medicina60030426)
Supplement: Supplementary file 1 [file medicina-60-00426-s001.zip › medicina-2829210-supplementary.pdf]

Table S1. Linear Regression Analyses for Path Design for Patients Diagnosed with Alzheimer's Disease.

Dependent Variables						
	CBI#		NPI##		SSF###	
	Adjusted Model		Adjusted Model		Adjusted Model	
Variables	β	p	β	p	β	p
Age≥80	.12	.18	.10	.23	.11	.19
Female	-.00	.50	-.09	.25	.23	.028
White	.32	.007	.08	.28	-.27	.014
MMSE≤17	.12	.16	.02	.44	----	----
ADL>7	.02	.43	.03	.09	----	----
NPI	.37	.002	----	----	----	----
CIRSG>7	-.01	.46	.02	.45	----	----
SSF	.12	.21	.33	.01	----	----

Notes:
N=66
p-values are 1-tailed.
#R^2adj=.19, F(8,57)=2.94, p=.008
##R^2adj=.-.00, F(7,58)=.96 p=.47
###R^2adj=.13, F(3,62)=4.21, p=.009.

Significant indirect effect found for SSF on CBI mediated through NPI: .33 x.37=.12; Sobel Test, z-score= 1.84, p=.03 (1-tailed).

Abbreviations: SFF= Subsyndromal/Syndromal Frailty; CBI=Caregiver Burden Interview; MMSE=Mini-Mental State Examination; ADL=Activities of Daily Living; NPI= Neuropsychiatric Inventory; CIRSG= Cumulative Illness Rating Scale Geriatrics
